# The Roxadustat (FG-4592) ameliorates tubulointerstitial fibrosis by promoting intact FGF23 cleavage

**DOI:** 10.1186/s12964-025-02175-2

**Published:** 2025-04-25

**Authors:** Jing Wang, Zuo-Lin Li, Yan Zhou, Zhong-Tang Li, Yan Tu, Xin-Hui Hu, Jin-Hua Zhu, Bi-Cheng Liu, Hong Liu

**Affiliations:** 1 Institute of Nephrology, Zhong da Hospital, Southeast University School of Medicine, Nanjing, Jiangsu 210009 China; 2 Department of Paediatrics, Zhong da Hospital, Southeast University School of Medicine, Nanjing, Jiangsu China; 3 Department of Nephrology, People’s Hospital of Yangzhong City, Zhenjiang, Jiangsu China

**Keywords:** Roxadustat, Tubulointerstitial fibrosis, FGF23, Anemia, Furin

## Abstract

**Background:**

Hypoxia-inducible factor prolyl hydroxylase inhibitor (HIF-PHI) represents a novel therapeutic approach for renal anemia, a prevalent complication of chronic kidney disease (CKD). However, the effects of HIF-PHI on renal functional outcomes remain poorly characterized. Here, the potential effects of FG-4592, an orally administered HIF-PHI, on renal fibrosis were explored systematically.

**Methods:**

In this study, a CKD rat model was established through subtotal 5/6 nephrectomy. Rats were administered either FG-4592 or vehicle control via oral gavage three times weekly for 12 consecutive weeks. Additionally, recombinant FGF23 was continuously delivered via subcutaneously implanted Alzet osmotic minipumps for 28 days.

**Results:**

Interestingly, we found that CKD-induced anemia was significantly ameliorated in CKD rats with FG-4592 treatment. Meanwhile, markedly alleviated histopathological changes and renal tubulointerstitial fibrosis (TIF) were observed in rats with FG-4592 administration. Notably, serum levels of intact FGF23 (iFGF23) were significantly reduced following FG-4592 administration in CKD rats. This finding was subsequently validated in CKD patients receiving Roxadustat therapy. Mechanistically, we illustrated that inhibition of the iFGF23-WNT5A pathway was the exact mechanism by which FG-4592 ameliorated TIF. Further, we also demonstrated that transcriptional activation of Furin enzyme was the exact molecular mechanism for FG-4592-mediated iFGF23 cleavage.

**Conclusions:**

FG-4592 attenuates TIF through Furin-mediated proteolytic cleavage of iFGF23. These findings provide novel mechanistic insights into HIF-PHI-mediated renal protection and establish a theoretical framework for clinical translation.

**Supplementary Information:**

The online version contains supplementary material available at 10.1186/s12964-025-02175-2.

## Introduction

Chronic kidney disease (CKD) affects approximately 13% of the global adult population, representing a significant public health challenge worldwide [1–4]. Anemia, one of the most frequent complications in CKD patients, is associated with substantial morbidity and adverse clinical outcomes [5, 6]. Novel pharmacological agents specifically targeting the underlying pathophysiology of anemia are currently under development, with the aim of improving therapeutic efficacy while minimizing treatment-related adverse effects.

Hypoxia-inducible factor prolyl hydroxylase domain inhibitors (HIF-PHIs) have emerged as a novel therapeutic approach for anemia management in CKD. Notably, Roxadustat, a recently approved orally administered and reversible HIF-PHI—has demonstrated favorable efficacy and safety profiles in treating renal anemia [7–9]. Mechanistically, HIF stabilization and subsequent erythropoiesis stimulation have been shown to occur in a dose-dependent manner [10]. While Roxadustat has demonstrated efficacy and tolerability in CKD patients with anemia, its long-term effects on renal function and fibrosis remain uncertain, particularly given the systemic exposure and pleiotropic biological consequences of sustained HIF activation, which may influence diverse pathological processes.

HIF-PHIs have been shown to modulate intact fibroblast growth factor 23 (iFGF23) levels [11]. FGF23, a phosphaturic hormone synthesized and secreted predominantly by osteocytes, exists in multiple isoforms. The mature bioactive form primarily functions to regulate renal phosphate excretion and vitamin D metabolism [12], circulating iFGF23 represents the full-length, biologically active isoform and is implicated in anemia progression, renal dysfunction, and mineral-bone disorders [13], In contrast, the biological role of C-terminal FGF23 (cFGF23) remains incompletely characterized. Preclinical studies in rodent models have demonstrated that cFGF23 fragments competitively inhibit FGF23-FGFR signaling pathways, thereby attenuating FGF23-mediated phosphaturic effects [14]. Emerging evidence further suggests that cFGF23 may possess anti-inflammatory properties and enhance iron utilization during acute inflammatory conditions [15, 16]. Notably, BAY 85–3934, a novel small-molecule oral HIF-PHI, has been reported to ameliorate anemia while reducing circulating FGF23 levels in a CKD murine model [17]. Nevertheless, the mechanistic basis for this association remains uncharacterized. Furthermore, clinical investigations have revealed parallel fluctuations between iFGF23 levels and serum phosphate concentrations following Roxadustat treatment [18]. These findings suggest that Roxadustat-mediated effects on renal function in CKD may be mechanistically linked to iFGF23 metabolic pathways.

This study investigated the dependency effect of FG-4592 on renal outcomes. Notably, therapeutic-dose FG-4592 administration was found to significantly attenuate tubulointerstitial fibrosis (TIF) in experimental models. Mechanistically, suppression of the iFGF23-WNT5A signaling axis constitutes the primary pathway through which FG-4592 mitigates TIF progression. Furthermore, transcriptional upregulation of the Furin protease was identified as the critical molecular mechanism governing FG-4592-induced iFGF23 proteolytic processing. These findings elucidate novel mechanisms underlying HIF-PHI-mediated renal protection while providing translational insights for optimizing anemia management strategies in CKD.

## Materials and methods

### Animals

All experimental procedures involving animals were approved by the Institutional Animal Care and Use Committee (IACUC) of Southeast University. Twenty-four male Sprague–Dawley rats (180 ~ 220 g) were randomly allocated into three experimental groups (*n* = 5 per group): sham-operated control, CKD + vehicle, and CKD + FG-4592 (MedChem Express, MCE, USA). All rats were maintained under standard laboratory conditions (22 ± 1 °C, 55 ± 5% humidity) with a 12-h light/dark cycle and ad libitum access to food and water. The CKD model was established through 5/6 nephrectomy (5/6 Nx) following the surgical protocol described by Yokoro et al. [19]. Sham-operated rats received two sequential laparotomies without renal tissue excision. CKD-induced rats were administered either FG-4592 (20 mg/kg) or vehicle control via oral gavage three times a week for 12 consecutive weeks [20]. FG-4592 was formulated as a micronized suspension (4 mg/mL) in distilled water containing 0.5% sodium carboxymethylcellulose (Na-CMC, Aladdin Biochemical Technology Co, Ltd, Shanghai, China). The vehicle control consisted of an aqueous solution containing 0.5% Na-CMC. The administered dose was adjusted according to individual body weight measurements obtained immediately prior to each treatment session.

On the 20th week after surgery, the rats were sacrificed and the blood samples obtained from the abdominal aorta were centrifuged at 3,000 rpm for 15 min to detect serum creatinine (Scr) and blood urea nitrogen (BUN) levels. Renal cortical tissues were used for pathological staining. The remaining samples were stored at − 80 °C for further experiments.

### Recombinant rat FGF23 intervention

High levels of FGF23 in normal rats were achieved by the infusion of rat rFGF23 (15 μg/d) for 28 days [21, 22]. CKD rats were surgically implanted with Alzet osmotic pumps (Durect Corporation, Cupertino, CA) as described previously [23] and infused with recombinant FGF23 (rFGF23) (MCE, USA) or vehicle (normal saline; VE) for 28 days. Specifically, Alzet pump model #2002 with a constant infusion rate of 0.5 μL/h over 28 days was used. Two experimental groups (*n* = 5 per group) were established: (1) CKD rats infused with FG-4592 and vehicle; (2) CKD rats infused with FG-4592 and rFGF23.

### Cell culture and treatments

Human renal tubular epithelial cell line HK-2 (American Type Culture Collection, USA) was cultured in Dulbecco’s Modified Eagle Medium–Ham’s F-12 medium (GE Healthcare, Waukesha, WI, USA) supplemented with 10% fetal bovine serum (FBS, Thermo Fisher Scientific, Waltham, MA, USA) in a 37 °C incubator with 5% CO_2_. HK-2 cells were transfected with Lipofectamine 3000 (Invitrogen) according to the manufacturer's protocol. In brief, cells were seeded in 6-well plates (2–3 × 10^5^ cells/well) and cultured to 60 ~ 80% confluence. The cells were treated with the indicated concentrations of FG-4592. Then, transforming growth factor-β1 (TGF-β1) (MCE) was added for 24 h (10 ng/ml) [24, 25]. Subsequently, rFGF23 was or vehicle (all from MCE) was added at the indicated concentrations for 24 h. The transfection complex was prepared according to the manufacturer’s instructions and added directly to the cells. The final concentration of Furin siRNA (sense 5′-CUGACCAAGUUCACUCUCGTT- 3′; antisense 5′- CGAGAGUGAACUUGGUCAGTT- 3′) or siNC were 100 nM. The final concentration of WNT5 A siRNA (sense 5′- CGCCCAGGUUGUAAUUGAATT- 3′; antisense 5′- UUCAAUUACAACCUGGGCGTT- 3′) or siNC were 100 nM.

UMR106 rat osteoblast-like cells (CRL- 1661; ATCC, Manassas, VA, USA) were cultured in Dulbecco’s Modified Eagle Medium (Gibco, Grand Island, NY, USA) with high glucose supplemented with 10% FBS, 100 U/ml penicillin, and 100 μg/ml streptomycin (Gibco, Life Technologies, Darmstadt, Germany). For experiments, 2 × 10^5^ cells per 6-well were used. To increase the low basal iFGF23 expression in these cells, UMR106 cells were pretreated with 10 nM 1,25(OH)_2_D_3_ (Merck, Darmstadt, Germany) for 24 h. Subsequently, 0.5 mM Indoxyl sulfate or vehicle (all from MCE) was added, and then stimulated with 50 μM FG-4592 or vehicle for 24 h. UMR- 106 cells were transfected with Lipofectamine 3000 (Invitrogen) according to the manufacturer’s protocol.

### Indirect co-culture by a Transwell system

Co-culture of UMR-106 cells and HK-2 cells was performed using a Transwell system. HK-2 cells were seeded into the lower compartment of the Transwell chamber (0.4 μm pore size, 24 mm diameter, Corning Costar, MA, USA), while UMR-106 cells were plated in the upper chamber (10^5^ cells/well). Cells were cultured separately for 24 h before co-culture conditions. FG-4592 was added to the upper UMR-106 cells, while the other group was treated with vehicle as a negative control group. Co-culture cells were maintained in DMEM with 10% FBS for 24 h. Subsequently, the HK-2 cells in the lower layer were collected for further studies.

### Analysis of physiologic parameters

The Scr, BUN, and hemoglobin levels were quantified using the commercial kits (Jiancheng, Nanjing, China). The iFGF23 concentration was examined on plasma collected from rats under fed conditions using ELISA assays specific for rat iFGF23 (Quidel, cat#: 60–6800). iFGF23 and cFGF23 concentration were examined on plasma collected from rats under fed conditions using ELISA assays specific for rat total FGF23 (Quidel, cat#: 60–6300). iFGF23 and cFGF23 concentration were examined on plasma collected from CKD patients under fed conditions using ELISA assays specific for human iFGF23 or total FGF23 (Quidel, cat#: 60–6600 and cat#: 60–6100). iFGF23 ELISAs detect full-length, biologically active FGF23 that can exert phosphaturia, whereas total FGF23 ELISAs detect both iFGF23 and its C-terminal fragments and therefore provide a measure of the total amount of circulating plasma FGF23.

### Histopathological assessment

Periodic Acid-Schiff (PAS) staining and Masson trichrome staining were performed to analyze kidney morphological changes. As previously described [26], kidney tissues were collected from rat, fixed with 4% buffered paraformaldehyde and then embedded in paraffin. PAS and Masson trichrome staining were performed on 4 μm-thick kidney sections according to the instructions of the PAS and Masson trichrome staining kits (Servicebio, China). The degree of tissue damage and TIF were scored according to the percentage of damaged tubules in PAS-stained sections and the stained tubulointerstitial area in Masson trichrome-stained sections, respectively, as previously described [26]. To evaluate kidney injury, images of the renal tubules were taken from 10 fields of Collagen 1 stained sections for each rat (200 × magnification).

### qPCR analysis

In this study, qPCR was performed as previously described by Li et al. [27]. Briefly, total RNA was extracted from the kidney superficial cortex or cultured cells, and then the RNA was reverse transcribed into cDNA using a cDNA Synthesis Kit (Vazyme Biotech Corporation, China) according to the instructions. qPCR was performed using SYBR premix Taq™ reagents (Vazyme Biotech Corporation) according to the instructions. Real-time quantitative PCR was performed on a 7300 PCR System (Thermo Fisher Scientific). Primers for real-time quantitative PCR are listed in Supplementary Table S1. Relative expression was normalized to glyceraldehyde 3-phosphate dehydrogenase levels.

### Chromatin immunoprecipitation assay

Chromatin immunoprecipitation (ChIP) assay was performed using the Simple ChIP Plus Enzymatic Chromatin IP Kit (Magnetic Beads, #9003, Cell Signaling Technology) according to previous descriptions [28]. Immunoprecipitation was performed with the antibody against HIF-1α (ab179483; Abcam) or normal IgG as the NC. Precipitated DNA fragments were detected by PCR using primers specific for the promoter region of Furin.

### Western blotting

Western blotting was performed as previously described by Liu et al. [29] Anti–HIF-1α (1:1000, Abcam, ab179483, USA), anti–alpha smooth muscle actin (α-SMA), (1:1000, Abcam, ab124964), anti-Collagen 1 (1:1000, Santa cruz, sc59772, USA), anti-fibronectin (1:1000, Abcam, ab45688), and anti–TGF-β1 (1:1000, Abcam, ab215715), anti-WNT5A (1:1000, Proteintech group, 55,184–1, China), anti-FGF23 (1:1000, Affinity Biosciences, DF3596, USA), anti-GAPDH (1:10,000, AF7021, Affinity Biosciences, USA), anti-β-catenin (1:1000, Affinity Biosciences, BF8016, USA), anti-β-actin (1:10000, Abways, AB2001, China), anti-Furin (1:1000, sc133142, Santa cruz), anti-rabbit IgG (1:3000, 7074S, Cell Signaling Technology, USA), and anti-mouse IgG (1:3000, 7076S, Cell Signaling Technology) antibodies were used.

### Immunohistochemistry

Immunohistochemistry was performed as previously described by Liu et al. [29]. Anti–HIF-1α (1:1000, Abcam, ab179483), anti–α-SMA, (1:1000, Abcam, ab124964), anti-Collagen 1 (1:1000, Santa cruz, sc59772), anti-fibronectin (1:1000, Abcam, ab45688), and anti–TGF-β1 (1:1000, Abcam, ab215715), anti-WNT5A (1:1000, Proteintech group, 55,184–1, China), anti-FGF23 (1:1000, Affinity Biosciences, DF3596, USA), anti-β-catenin (1:1000, Affinity Biosciences, BF8016, USA), and anti-Furin (1:1000, sc133142, Santa cruz), antibodies were used.

### Clinical study

This is a single-center retrospective study investigating Roxadustat versus Recombinant Human Erythropoietin (rHuEPO) in the management of anemia among patients with CKD. This study included non-dialysis patients with CKD stages 3–5 who were hospitalized at Zhongda Hospital affiliated to Southeast University. All patients provided written, informed consent to participate in this study. Recruited patients were screened according to inclusion and exclusion criteria (Supplementary Fig. 4). The inclusion criteria were as follows: (a) age over 18 and under 70; (b) CKD 3–5 stage patients with Hgb levels of > 90 g/l and < 120 g/l; (c) the patient has not been treated with Roxadustat. The exclusion criteria were as follows: (a) body weight greater than 110 kg or less than 45 kg. (b) diagnosed with active hepatitis or liver failure; (c) uncontrolled hypertension; (d) a history of tumors and radiation therapy or chemotherapy in the past 6 months; (e) anemia unrelated to CKD; (f) pregnant or lactating women. We enrolled two patient groups (Roxadustat-treated and rHuEPO-treated), The starting dose of Roxadustat was either 70 mg (body weight 45–60 kg) or 100 mg (body weight of 60 kg or greater). Participants in the rHuEPO group received subcutaneous injections of rHuEPO at a dosage range of 50–100 IU/kg body weight, administered in 1 to 3 divided doses per week.

Routine biochemical parameters and levels of Hgb, Creatinine, iFGF23 and total FGF23 were measured in venous blood samples obtained at baseline, and no statistically significant differences in baseline characteristics were observed between the groups (*P* > 0.05 for all covariates). Levels of Hgb, Creatinine, iFGF23 and total FGF23 were measured in venous blood samples at 4 and 12 weeks thereafter.

### Statistical analysis

Data are expressed as mean ± standard deviation (SD). For experiments comparing 2 groups, the results were analyzed using the Student’s *t*-test or *Mann–Whitney U* test. For analysis of more than two groups one-way analysis of variance (ANOVA) followed by Dunnett’s or Bonferroni’s multiple comparisons test was applied. A *P* value < 0.05 means that the difference was statistically significant. Statistical analysis was performed using GraphPad Prism 10 (Version 10.0.1; GraphPad Software Inc.).

## Result

### FG-4592 ameliorates TIF in CKD rats

**Fig. 1:**
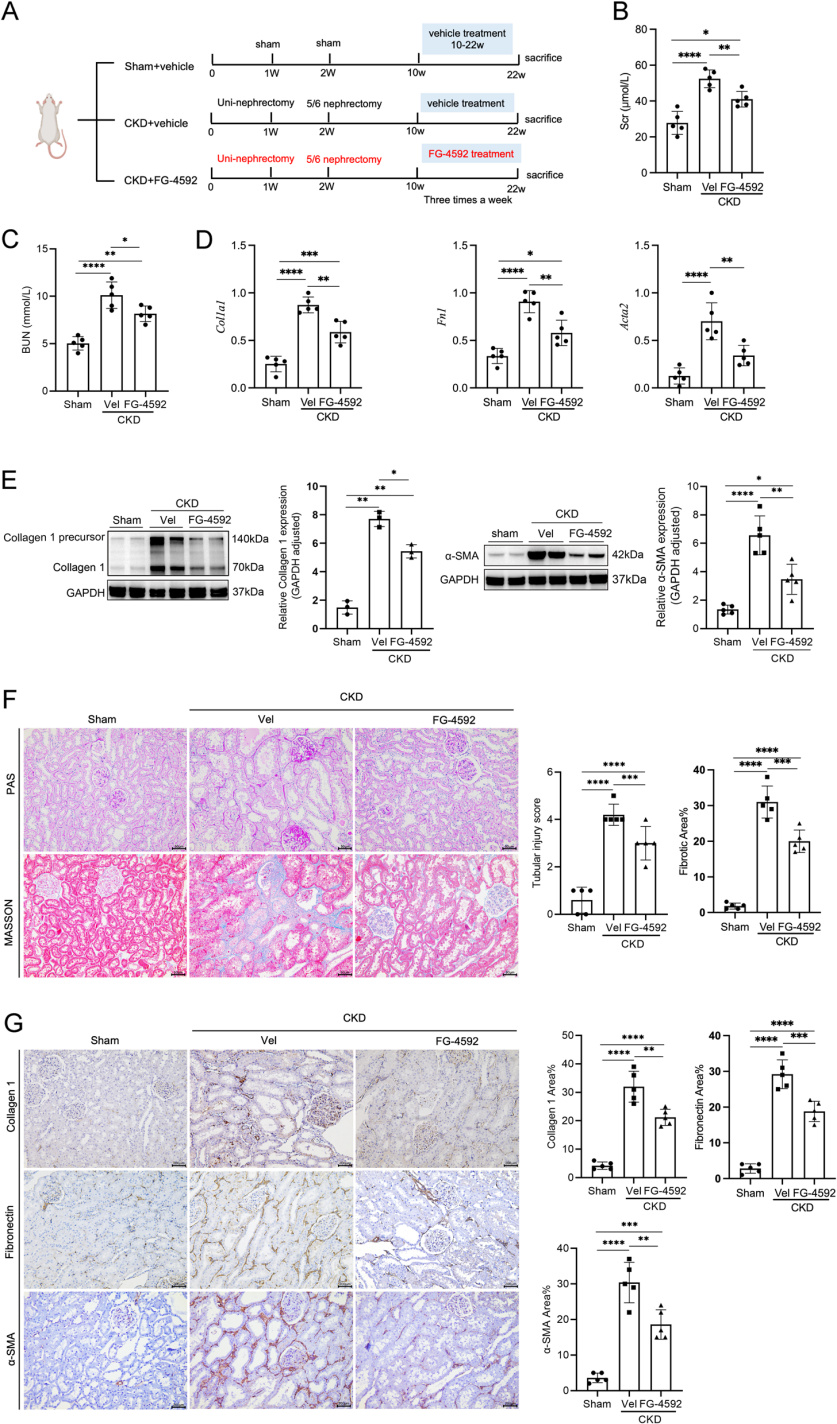
FG-4592 ameliorates TIF in CKD rats. Parameters measured include (**A**) Schematic diagram illustrating the experimental groups of rats: Sham, CKD + vehicle, and CKD + FG-4592. Rats in the sham-surgery group underwent two laparotomy procedures without tissue removal. The 5/6 Nx model was employed to establish CKD. CKD rats were administered FG-4592 (20 mg/kg) or vehicle control via oral gavage for 12 weeks. Kidney tissues were collected 12 weeks post-treatment with FG-4592 or vehicle. **B** and **C** Renal function across the three groups was assessed by measuring Scr and BUN levels. **D** *Col1a1*, *Fn1*, and *Acta2* mRNA expression of three groups of rats in the cortex of kidney, as determined by real-time PCR. **E** Protein expression levels of Collagen 1 and α-SMA in the renal cortex were determined by Western blotting. GAPDH (glyceraldehyde 3-phosphate dehydrogenase) served as the loading control. **F** Representative images of PAS staining and fibrotic area percentage in kidney tissues. Scale bars: 50 μm. **G** Representative immunohistochemical images showing Collagen 1 and α-SMA expression in kidney tissues following FG- 4592 or vehicle treatment. Scale bars: 200 μm. Data are presented as means ± SD (*n* = 5). *p < 0.05, ** *p* < 0.01, *** *p* < 0.001, **** *p* < 0.0001

To investigate the therapeutic potential of FG-4592 in CKD, we established an adenine-induced rat CKD model and administered either FG-4592 (20 mg/kg) or vehicle control via daily oral gavage for 12 weeks (Fig. 1A). Initially, we evaluated changes in body weight and hemoglobin levels after 12 weeks of FG-4592 treatment. As anticipated, CKD rats exhibited moderate anemia and significant weight loss; however, FG-4592 administration markedly corrected anemia and mitigated weight loss in rats (Supplementary Fig. 1 A-B). Additionally, compared to vehicle-treated CKD rats, those receiving FG-4592 showed significant reductions in Scr and BUN levels, suggesting that FG-4592 treatment improved renal function in CKD rats (Fig. 1B-C). Histological assessment of TIF using Masson’s trichrome staining revealed moderate TIF in CKD rats, which was significantly alleviated by FG-4592 treatment (Fig. 1F). Furthermore, we analyzed the mRNA and protein expression levels of key markers associated with TIF. Notably, the mRNA levels of *Fn1*, *Col1a1*, and *Acta2* in the kidneys of CKD rats were significantly elevated but markedly downregulated following FG-4592 treatment (Fig. 1D). This pattern was corroborated by western blot analysis (Fig. 1E). Consistent with these findings, IHC staining demonstrated that FG-4592 significantly suppressed the expression of α-SMA and Collagen 1 in renal tissues (Fig. 1G). Moreover, Scr and BUN levels were positively correlated with the expression of Collagen 1, Fibronectin, and α-SMA in the kidneys of CKD rats, respectively (Supplementary Fig. 2 A-F). Taken together, these results demonstrate that FG-4592 improves renal function and attenuates TIF in CKD rats.

### FG- 4592 lowers iFGF23 level in CKD rats and CKD patients

**Fig. 2:**
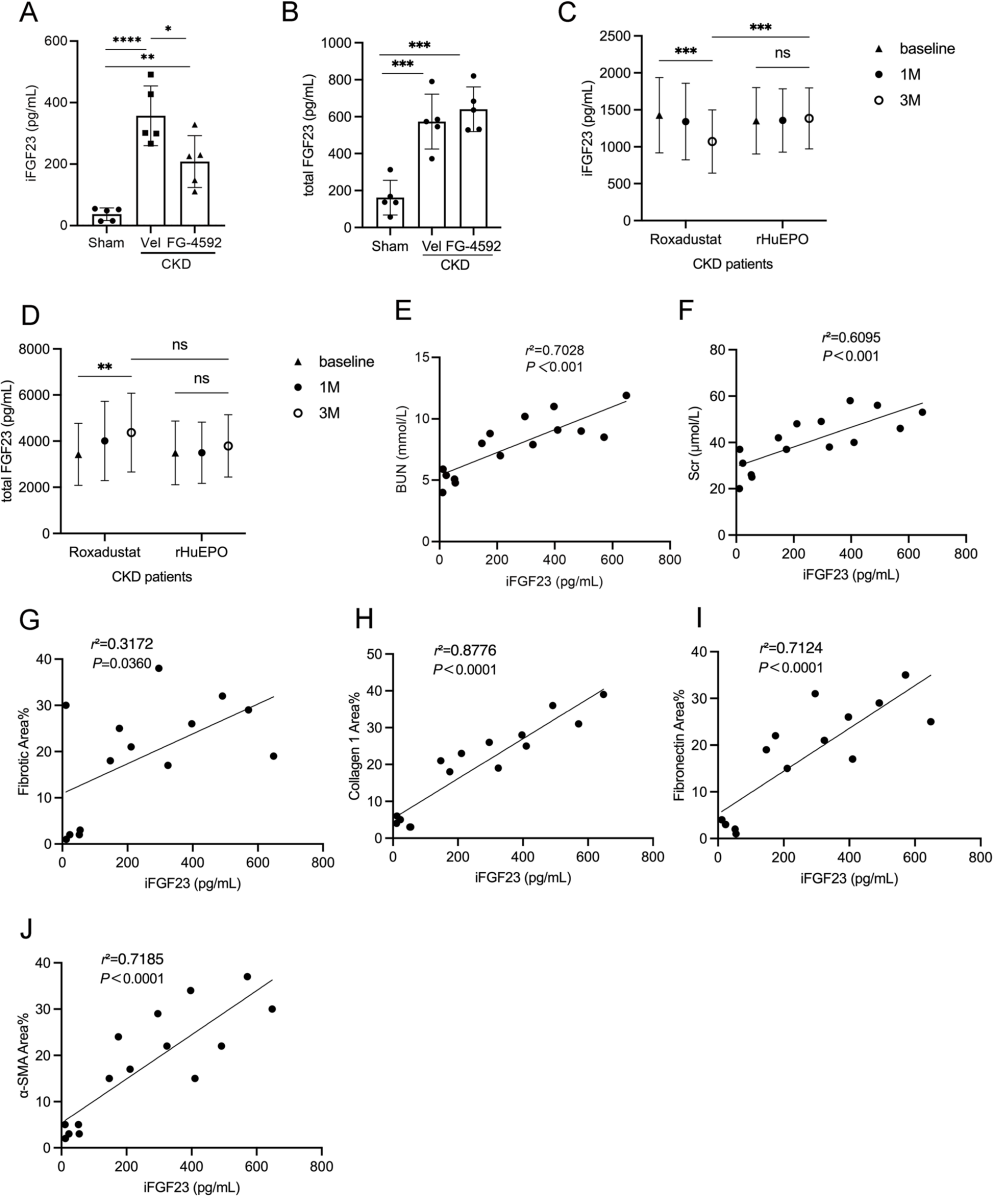
FG-4592 lowers iFGF23 level in CKD rats and CKD patients. Parameters measured include (**A**) Serum intact fibroblast growth factor 23 (iFGF23) levels in CKD rats. **B** Serum total FGF23 levels in CKD rats. **C** Serum iFGF23 levels in CKD patients. **D** Serum total FGF23 levels in CKD patients. **E** Scatter plots with linear regression analysis demonstrating a significant correlation between blood urea nitrogen (BUN) and iFGF23 levels in CKD rats. **F** Scatter plots with linear regression analysis showing a significant correlation between Scr and iFGF23 levels in CKD rats. **G** Scatter plots with linear regression analysis revealing a significant correlation between iFGF23 levels and the percentage of fibrotic area in the kidney of CKD rats. **H** Scatter plots with linear regression analysis indicating a significant correlation between iFGF23 levels and Collagen 1 staining intensity in the kidney of CKD rats. **I** Scatter plots with linear regression analysis illustrating a significant correlation between iFGF23 levels and Fibronectin staining intensity in the kidney of CKD rats. **J** Scatter plots with linear regression analysis demonstrating a significant correlation between iFGF23 levels and α-SMA staining intensity in the kidney of CKD rats. Data are presented as means ± SD (*n* = 5). Statistical significance is denoted as follows: *p < 0.05, **p < 0.01, ***p < 0.001, ****p < 0.0001

The circulating iFGF23 levels were measured using an iFGF23 ELISA kit. Our results demonstrated that the serum iFGF23 concentration was significantly elevated in CKD rats compared to sham-operated controls. Additionally, we observed that FG-4592 treatment effectively reduced serum iFGF23 levels in CKD rats (Fig. 2A-B). To further validate these findings, we conducted a clinical study comparing FG-4592 (roxadustat) with recombinant human erythropoietin (rHuEPO) in treating renal anemia. Baseline patient characteristics are provided in Supplementary Table 2. After three months of treatment, (Fig. 2C-D). HgB levels showed no statistically significant difference between the Roxadustat group and rHuEPO group *(P* > 0.05). However, the Roxadustat group showed a significant decrease in iFGF23 levels and a reduction in creatinine levels compared to the rHuEPO group *(P* < 0.05) (Supplementary Fig. 3 A-B; Supplementary Table 3–6). Importantly, a positive correlation was identified between iFGF23 levels and both Scr and BUN levels (Fig. 2E-F). Furthermore, iFGF23 levels were positively correlated with the fibrotic area percentage in the kidneys of CKD rats (Fig. 2G), as well as with the expression of Collagen 1, Fibronectin, and α-SMA in renal tissues. Collectively, these findings suggest that FG-4592 reduces circulating iFGF23 levels in both rat model of CKD and CKD patients, and that iFGF23 is closely associated with TIF.

### The renoprotective effects of FG-4592 were abrogated by FGF23 overexpression

**Fig. 3:**
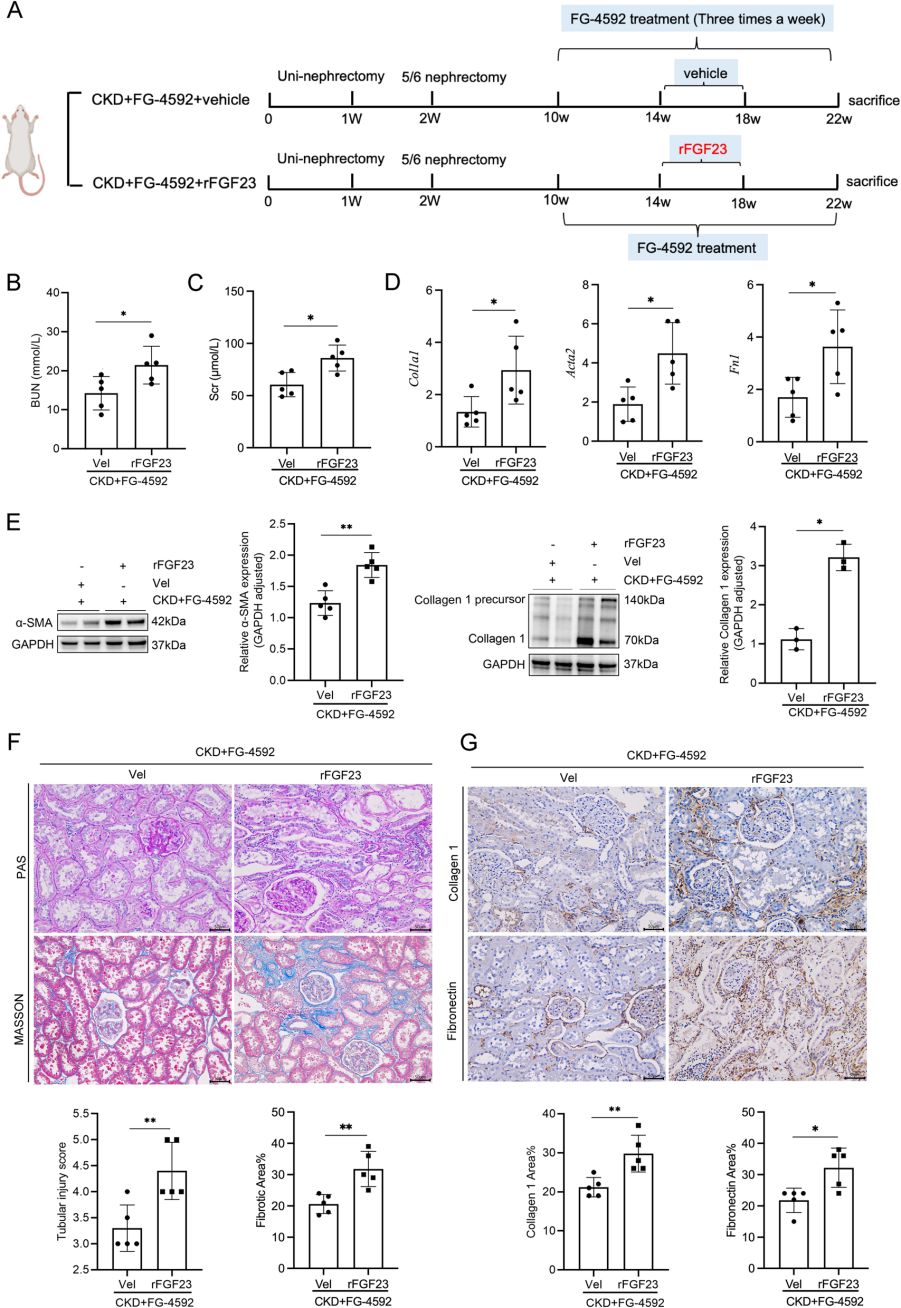
The renoprotective effects of FG-4592 were abrogated by FGF23 overexpression. Parameters measured include (**A**) Schematic diagram illustrating the experimental groups of rats: CKD + FG-4592 + vehicle and CKD + FG-4592 + rFGF23. **B** and **C** Renal function was assessed by measuring Scr and BUN levels. **D** mRNA expression levels of *Col1a1*, *Fn1*, and *Acta2* in the renal cortex were quantified using real-time PCR. **E** Western blot analysis was performed to evaluate the protein expression of Collagen 1 and α-SMA, which reflect the extent of TIF. GAPDH (glyceraldehyde 3-phosphate dehydrogenase) was used as the loading control. **F** Representative images of PAS staining and the percentage of fibrotic area in kidney tissues. Scale bars: 50 μm. **G** Representative immunohistochemical images demonstrating the upregulation of Collagen 1 and α-SMA in the CKD + FG-4592 + rFGF23 group. Scale bars: 50 μm. Data are presented as means ± SD (*n* = 5). Statistical significance is denoted as follows: *p < 0.05, **p < 0.01, ***p < 0.001, ****p < 0.0001

Subcutaneous implantation of osmotic minipumps was employed to deliver recombinant FGF23 (rFGF23) in CKD rats for 4 weeks, successfully inducing sustained rFGF23 overexpression (Fig. 3A). Biochemical analysis revealed that rFGF23 overexpression significantly elevated Scr and BUN levels (Fig. 3B-C). Histopathological evaluation using Masson’s trichrome staining demonstrated significant exacerbation of TIF in rFGF23-overexpressing CKD rats (Fig. 3F). Quantitative RT-PCR analysis confirmed significant upregulation of fibrotic markers *Fn1*, *Col1a1*, and *Acta2* mRNA in renal tissue following rFGF23 overexpression (Fig. 3D). Western blot analysis parallelly demonstrated increased expression of fibrosis-related proteins collagen 1 and α-smooth muscle actin (α-SMA) in the rFGF23 overexpression group (Fig. 3E). Immunohistochemical staining of renal fibrosis biomarkers yielded concordant results (Fig. 3G). Collectively, these findings demonstrate that rFGF23 overexpression abolishes the renoprotective effects of FG-4592.

### iFGF23 mediates FG-4592-induced inhibition of tubular cell fibrogenesis

**Fig. 4:**
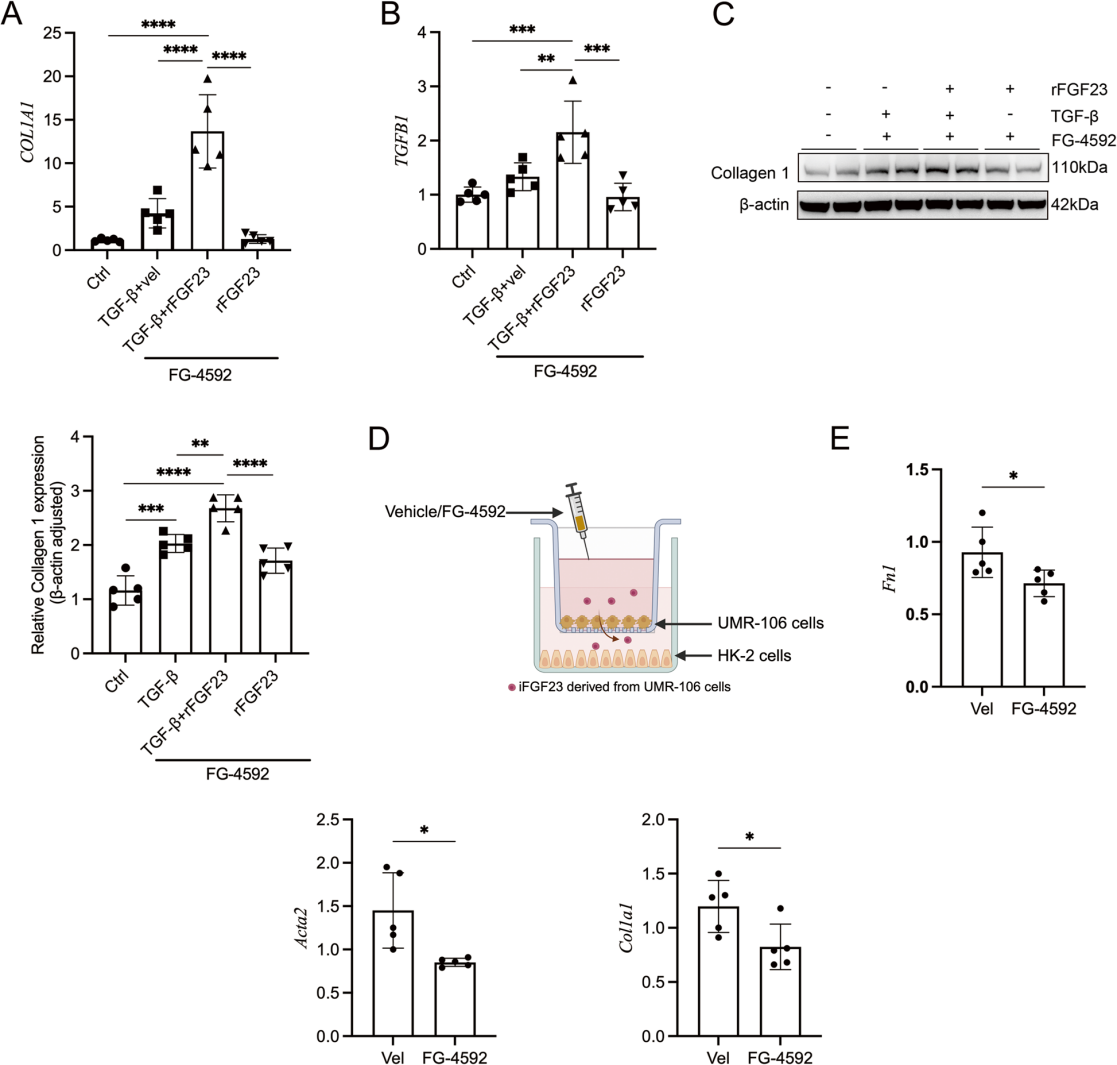
iFGF23 mediates FG-4592-induced inhibition of tubular cell fibrogenesis. HK-2 cells were pre-treated with TGF-β1 (10 ng/mL) for 24 h, followed by treatment with recombinant FGF23 (rFGF23, 50 ng/mL) or vehicle for an additional 24 h. Parameters measured include: **A** *COL1A1* mRNA expression levels in HK-2 cells, as determined by quantitative PCR (qPCR). **B** *TGFB1* mRNA expression levels in HK-2 cells, as determined by qPCR. **C** Collagen 1 protein expression levels in HK-2 cells, as assessed by Western blot analysis. **D** Schematic illustration of the co-culture system using UMR-106 cells and HK-2 cells in a Transwell setup. **E** mRNA expression levels of *Fn1*, *Acta2*, and *Col1a1* in HK-2 cells, as quantified by qPCR. Data are presented as means ± SD (*n* = 5). Statistical significance is denoted as follows: *p < 0.05, **p < 0.01, ***p < 0.001, ****p < 0.0001

Considering that tubular epithelial cells serve as crucial mediators of interstitial inflammation and fibrosis [30], we hypothesized that these cells represent the primary effectors of iFGF23-mediated FG-4592 actions. To investigate the fibrogenic potential of iFGF23, human proximal tubular epithelial cells (HK-2) were treated with 50 μM FG- 4592. Following TGF-β1 stimulation (24 h), cells were treated with 50 ng/ml recombinant FGF23 (rFGF23) or vehicle control. Notably, rFGF23 treatment significantly upregulated *Col1a1* and *TGFB1* mRNA expression (Fig. 4A-B). Western blot analysis confirmed corresponding upregulation of Collagen I at the protein level (Fig. 4C). To examine osteoblast-tubular epithelial cell interactions, a Transwell co-culture system was established using UMR-106 osteoblast-like cells and HK-2 cells (Fig. 4D). FG-4592 was administered to UMR-106 cells in the upper chamber, with vehicle-treated cells serving as negative controls. Following treatment, HK-2 cells (subjected to TGF-β1 intervention) from the lower chamber were harvested for gene expression analysis. Quantitative PCR analysis of harvested HK-2 cells demonstrated significant downregulation of *Fn1*, *Acta2*, and *Col1a1* mRNA expression. FG-4592 treatment significantly reduced Fn1, Acta2, and *Col1a1* expression compared to vehicle-treated controls (Fig. 4E). These data indicate that iFGF23 was involved in FG-4592-induced inhibition of tubular epithelial cell fibrogenesis.

### FG- 4592 ameliorates TIF by inhibiting iFGF23-WNT5A pathway

**Fig. 5:**
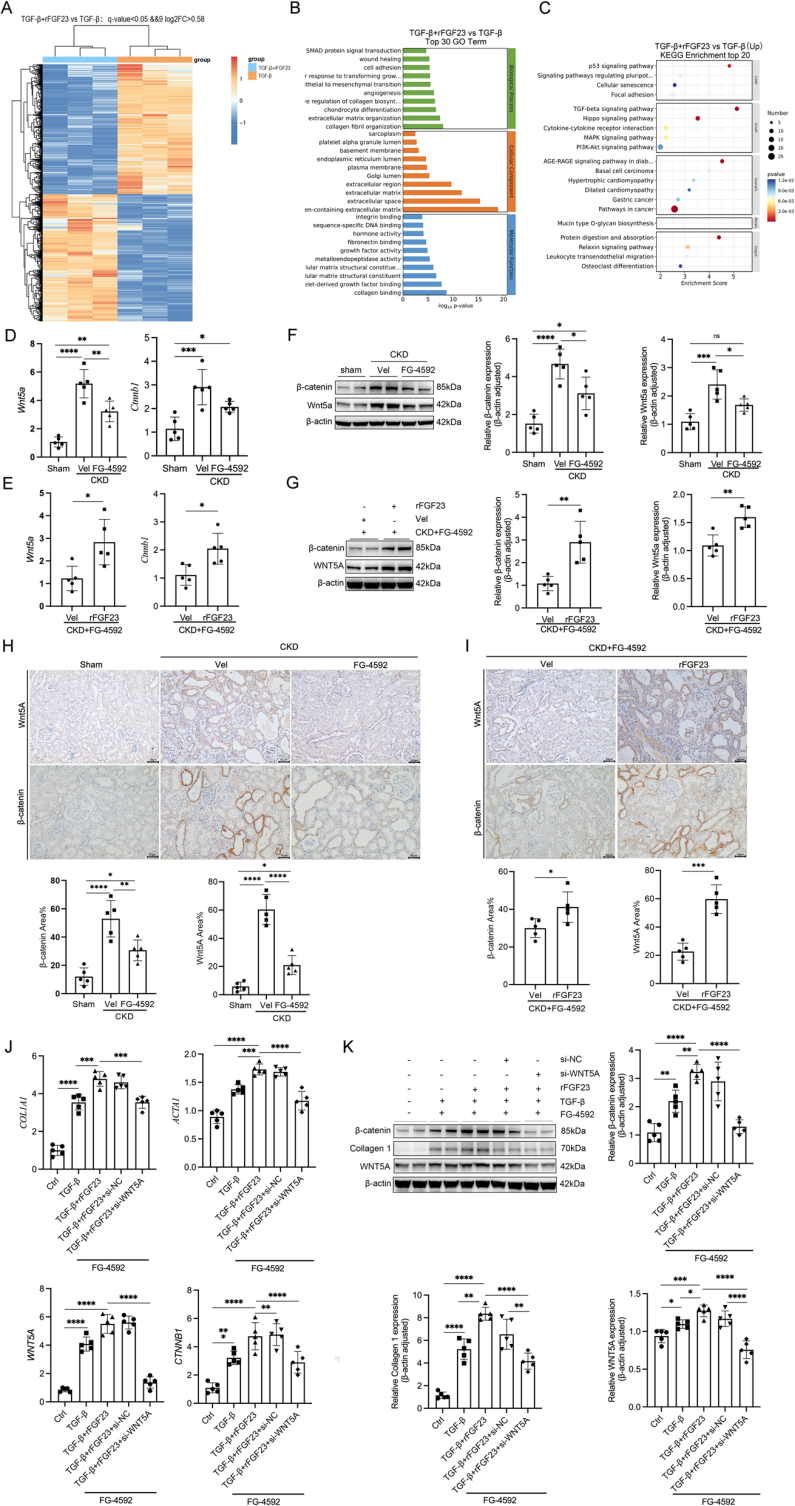
FG-4592 ameliorates TIF by inhibiting iFGF23-WNT5A pathway. Parameters measured include (**A**) Hierarchical clustering analysis revealing distinct mRNA expression profiles among the experimental groups. Yellow and blue shades indicate expression levels above and below the relative mean, respectively, across all samples. **B** Gene Ontology (GO) enrichment analysis. **C** Kyoto Encyclopedia of Genes and Genomes (KEGG) pathway analysis. **D** and **F** mRNA and protein expression levels of Wnt5a and β-catenin in the following groups: sham, CKD + vehicle, and CKD + FG-4592. **E** and **G** mRNA and protein expression levels of Wnt5a and β-catenin in the following groups: CKD + FG-4592 + vehicle and CKD + FG-4592 + rFGF23. **H** and **I** Representative immunohistochemical images showing Wnt5a and β-catenin expression in kidney tissues across different experimental groups. **J** mRNA expression levels of *COL1A1*, *ACTA1*, *WNT5A*, and *CTNNB1* in HK-2 cells following WNT5A siRNA interference, as determined by quantitative PCR (qPCR). **K** Protein expression levels of Collagen 1, Wnt5a, and β-catenin in HK-2 cells after WNT5A siRNA interference, as assessed by Western blot analysis. Scale bars: 50 μm. Data are presented as means ± SD (*n* = 5). Statistical significance is denoted as follows: *p < 0.05, **p < 0.01, ***p < 0.001, ****p < 0.0001

To elucidate the precise mechanisms underlying FG-4592-mediated attenuation of TIF via iFGF23 regulation, we conducted genome-wide transcriptomic analysis in target cells. Differentially expressed genes were visualized using a volcano plot (Fig. 5A). Gene Ontology (GO) and Kyoto Encyclopedia of Genes and Genomes (KEGG) pathway analyses were performed to characterize transcriptomic profiles and predict functional annotations of differentially expressed genes (Fig. 5B-C). Genes with significant differential expression (false discovery rate < 0.05) in cluster 4 (Fig. 5A) were subjected to pathway enrichment analysis to identify fibrogenesis-related regulatory networks. Pathway analysis revealed significant enrichment of fibrogenesis-related pathways (Fig. 5A-B), suggesting a potential profibrotic role of iFGF23 in tubular epithelial cells (TECs).

RNA sequencing analysis identified significant upregulation of WNT5A, a key fibrotic regulator, in HK-2 cells co-stimulated with rFGF23 and TGF-β1. Quantitative RT-PCR and Western blot analyses were performed to assess WNT5A and β-catenin expression at both mRNA and protein levels in CKD rats. Notably, FG-4592 treatment significantly reduced WNT5A and β-catenin expression compared to vehicle-treated CKD rats (Fig. 5D-E). Conversely, rFGF23 overexpression significantly upregulated WNT5A and β-catenin expression compared to vehicle controls (Fig. 5F-G). Immunohistochemical staining confirmed these findings for WNT5A and β-catenin expression patterns (Fig. 5H-I). In HK-2 cells, combined rFGF23 and TGF-β1 stimulation significantly upregulated WNT5A and β-catenin expression compared to TGF-β1 stimulation alone (Fig. 5J-K).

To investigate the functional role of WNT5A in TEC fibrogenesis, siRNA-mediated WNT5A knockdown experiments were conducted. WNT5 A siRNA transfection significantly reduced WNT5A protein levels in HK-2 cells co-stimulated with rFGF23 and TGF-β1. WNT5 A knockdown significantly attenuated the upregulation of α-smooth muscle actin (α-SMA) and collagen 1 (Fig. 5J-K). These findings demonstrate that FG-4592 attenuates TIF through downregulation of the iFGF23-WNT5A signaling pathway.

### FG-4592 lowers iFGF23 levels through Furin-mediated cleavage

**Fig. 6:**
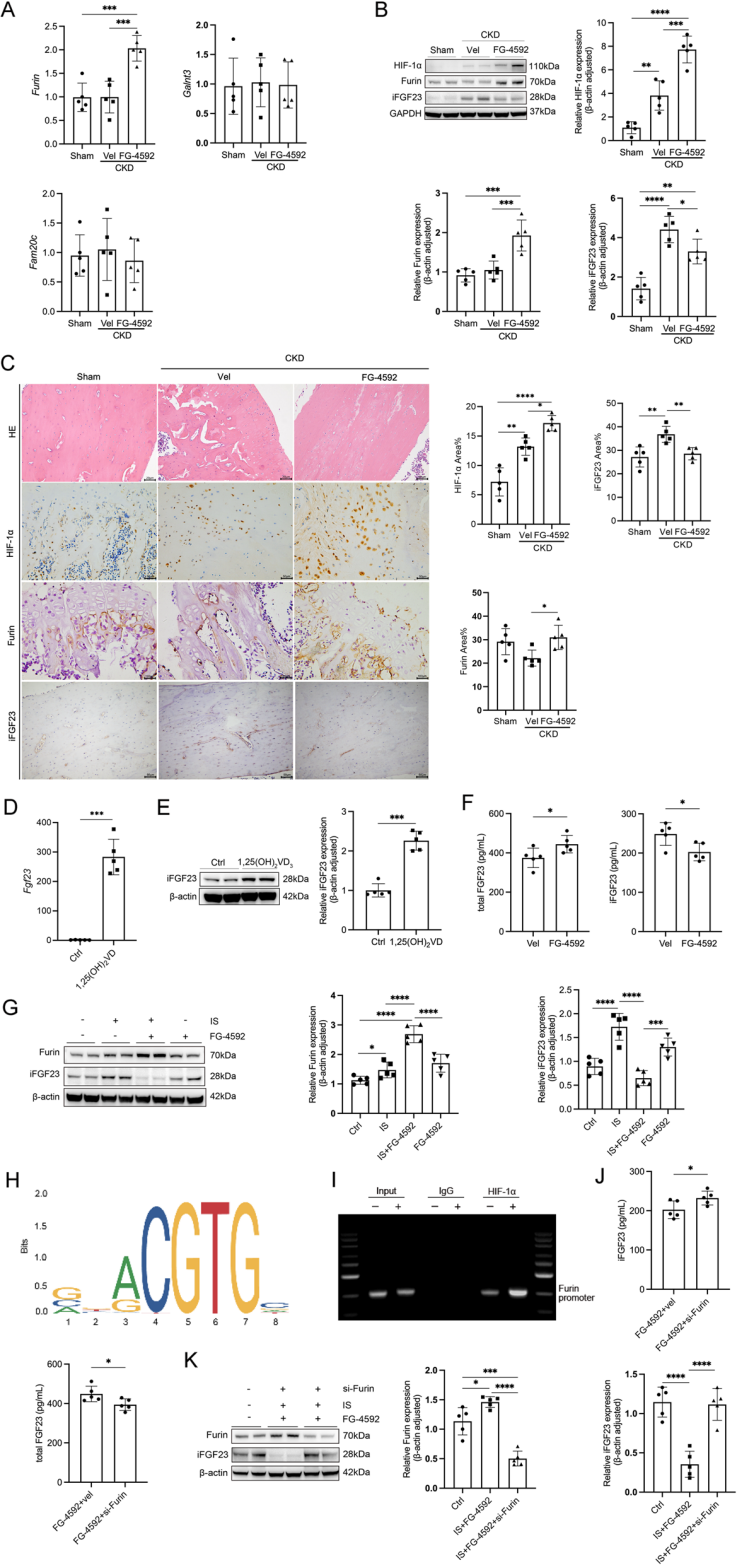
FG-4592 lowers iFGF23 levels through Furin-mediated cleavage. Parameters measured include (**A**) mRNA expression levels of *Furin*, *Galnt3*, and *Fam20c* in bone tissues from the following groups: sham, CKD + vehicle, and CKD + FG-4592, as determined by quantitative PCR (qPCR). **B** Protein levels of HIF-1α, Furin, and intact fibroblast growth factor 23 (iFGF23) in bone tissues treated with FG-4592 or vehicle, as assessed by Western blot analysis. **C** Representative images of hematoxylin and eosin (HE) staining of bone tissues (scale bars: 20 μm), along with IHC staining for HIF-1α and iFGF23 (scale bars: 50 μm) and Furin (scale bars: 100 μm). **D** UMR-106 cells were pretreated with 10 nM 1,25(OH)_2_D_3_, and the expression of FGF23 mRNA was analyzed using real-time PCR. **E** Western blot analysis was performed to measure iFGF23 protein expression in UMR-106 cells. Cells were pretreated with indoxyl sulfate (IS, 0.5 mM) or vehicle, followed by treatment with FG-4592 (50 μM) or vehicle for 24 h. **F** Levels of iFGF23 and total FGF23 in the cell supernatant were quantified using an ELISA assay kit. **G** Western blot analysis was used to evaluate the protein expression of iFGF23 and Furin in UMR-106 cells. **H** Motif map of the Furin promoter region, highlighting the HIF-1 binding site. **I** ChIP assays demonstrated HIF-1 binding to the Furin promoter, which was enhanced by FG-4592 stimulation (50 μM for 24 h). **J** Protein expression levels of Furin and iFGF23 in UMR-106 cells following Furin siRNA interference, as determined by Western blot analysis. **K** Levels of iFGF23 and total FGF23 in the cell supernatant were measured using an ELISA assay kit after Furin siRNA interference. Data are presented as means ± SD (*n* = 5). Statistical significance is denoted as follows: *p < 0.05, **p < 0.01, ***p < 0.001, ****p < 0.0001

Finally, we investigated the precise mechanism by which FG-4592 reduces iFGF23 levels. It is well established that three key enzymes—Furin, the family with sequence similarity 20 C (FAM20 C), and polypeptide N-acetylgalactosaminyltransferase 3 (GALNT3)—play critical roles in regulating iFGF23 metabolism. Interestingly, we observed an approximately twofold increase in *Furin* mRNA levels in the bones of CKD rats following FG-4592 treatment, while no significant changes were detected in the expression of *GALNT3* and *FAM20 C* (Fig. 6A). Consistent with these findings, Furin protein expression in the bones of CKD rats was also upregulated after FG-4592 treatment, as confirmed by western blotting (Fig. 6B) and immunohistochemical staining (Fig. 6C). To further explore whether FG-4592 modulates Furin expression—a key protease involved in iFGF23 cleavage—in bone cells, we conducted experiments using UMR-106 cells. Initially, UMR-106 cells were pretreated with 10 nM 1,25(OH)_2_D_3_ for 24 h to elevate the low basal levels of iFGF23 RNA and protein (Fig. 6D-E). Subsequent experiments revealed that FG-4592 treatment downregulated iFGF23 levels in the cell supernatant while increasing the levels of cFGF23 (Fig. 6F). Additionally, FG-4592 stimulated *Furin* expression and reduced iFGF23 levels in UMR-106 cells (Fig. 6G). To elucidate the transcriptional regulation of *Furin*, we utilized the JASPAR database to predict potential binding sites for HIF-1α within the *Furin* promoter region (Fig. 6H). ChIP assays confirmed that HIF-1α did not bind to the *Furin* promoter under basal conditions; however, HIF-1α binding was significantly enriched at the promoter following FG-4592 stimulation (50 μM for 24 h) in UMR-106 cells (Fig. 6I). These results demonstrate a direct interaction between HIF-1α and the *Furin* promoter in response to FG-4592 treatment, highlighting HIF-1α as a strong positive regulator of *Furin* gene expression during FG-4592 administration.

Then, to further characterize Furin’s potential functional role in iFGF23 cleavage, knockdown experiments were conducted using siRNA targeting Furin. An increase in iFGF23 levels accompanied by a decrease in cFGF23 levels was observed in the supernatant of UMR-106 cells transfected with Furin siRNA (Fig. 6J). Furin protein expression was significantly reduced in indoxyl sulfate-stimulated cells following siRNA transfection. Furthermore, Furin knockdown significantly attenuated iFGF23 cleavage in indoxyl sulfate-stimulated cells (Fig. 6K). These findings demonstrate Furin's critical involvement in iFGF23 processing during FG-4592 treatment.

## Discussion

HIF-PHIs represent a novel class of oral agents utilized for the treatment of renal anemia through the stabilization of HIF. Given the pleiotropic effects associated with HIF activation, the impact of HIF-PHIs on renal function remains poorly understood. In this study, the potential effects of Roxadustat (FG-4592), a first-in-class HIF-PHI employed for the treatment of renal anemia, on renal fibrosis were investigated. Notably, it was observed that FG-4592 attenuated TIF through the promotion of iFGF23 cleavage, a process mediated by Furin protease. The findings not only offer novel insights into the effects of FG-4592 on renal function but also establish a significant theoretical foundation for clinical practice.

Given the pleiotropic effects of the HIF pathway, a master regulator of various biological processes, concerns have been raised that Roxadustat treatment for anemia may exacerbate CKD [10]. Indeed, a phase 3 trial of Roxadustat indicated that, compared to placebo, Roxadustat treatment for anemia was associated with a greater decline in kidney function. Specifically, the annual rate of change in estimated glomerular filtration rate was − 3.70 mL/min/1.73 m[2] with Roxadustat, compared to − 3.19 mL/min/1.73 m^2^ with placebo (*P* = 0.046) [31]. For clinical research, we compared the therapeutic efficacy of Roxadustat versus rHuEPO in managing renal anemia among non-dialysis patients with stage 3 ~ 5 CKD. After three months of treatment, while Roxadustat demonstrated no statistically significant difference in improving anemia compared to rHuEPO. it exhibited unique advantages, iFGF23 Reduction: The roxadustat group showed a significant decrease in intact fibroblast growth factor 23 (iFGF23) levels (*P* < 0.01 vs. rHuEPO group). Renal Function Preservation: Roxadustat-treated patients had lower serum creatinine levels (323.7 ± 58.2) compared to rHuEPO recipients (361.6 ± 68.8, *P* < 0.05). These findings suggest that Roxadustat may exert renoprotective effects through mechanisms independent of its anemia-correcting role. Therefore, it is critically important to evaluate the impact of Roxadustat on the progression of kidney disease. In this study, it was demonstrated that FG-4592 significantly ameliorates TIF in CKD rats, consistent with previous findings. For example, Naito et al. demonstrated that Roxadustat (FG-4592) reduces renal fibrosis in Dahl salt-sensitive rats. However, the precise regulatory mechanisms underlying these effects remain to be elucidated.

Previous clinical evidence suggested that HIF-PHIs may influence FGF23 [18], a phosphaturic hormone primarily derived from and secreted by bone osteocytes. Mature, bioactive FGF23 primarily targets the kidney to regulate phosphate and vitamin D homeostasis [12], a process closely linked to phosphate metabolism, inflammation, and erythropoiesis. Emerging evidence demonstrated that anemia and disturbances in iFGF23 homeostasis are interrelated complications of CKD [32]. Notably, disturbances in FGF23 homeostasis are associated with CKD progression, cardiovascular disease, and mortality [13, 33]. Therefore, it is plausible to hypothesize that iFGF23 plays a crucial role in mediating the effects of FG-4592 on renal function. Notably, it was observed that therapeutic doses of FG-4592 reduce iFGF23 levels in CKD rats. Furthermore, similar results were observed in patients treated with Roxadustat. Subsequently, overexpression of rFGF23 was found to reverse the protective effects of FG-4592 on the kidney. Thus, the protective effects of FG-4592 on TIF were found to be iFGF23-dependent, providing novel insights into the pathogenesis of CKD.

Then, the precise mechanism through which iFGF23 deficiency mediates TIF attenuation during FG-4592 treatment remains to be fully elucidated. Previous studies have demonstrated that FGF23, FGF receptors, and the obligate co-receptor alpha-Klotho act in concert to mediate FGF23 actions on target organs, including the kidney. However, accumulating evidence suggests that iFGF23 can also target cells in a Klotho-independent manner, particularly under conditions of elevated FGF23 concentrations [34]. Therefore, we hypothesize that iFGF23 deficiency contributes to TIF alleviation by suppressing specific target genes. Notably, mRNA sequencing revealed a significant upregulation of WNT5A in tubular cells stimulated with rFGF23 and TGF-β1. Furthermore, significant induction of WNT5A mRNA and protein levels was observed in the rFGF23 overexpression group in vivo. Consistent with our findings, Muñoz-Castañeda et al. reviewed the relationship between the FGF23/Klotho axis and the Wnt/β-catenin pathway [35]. Thus, inhibition of the iFGF23-WNT5A pathway represents a key molecular response of the kidney to FG-4592 treatment. To our knowledge, this study is the first to characterize the role of the iFGF23-WNT5 A pathway in the context of kidney exposure to FG-4592, providing novel insights into CKD pathophysiology. Another critical question is the precise mechanism through which FG-4592 reduces iFGF23 levels in CKD.

It is previously recognized that coordinated regulation of FGF23 transcription and proteolytic processing generates dynamic circulating ratios of iFGF23, N-terminal fragments, and cFGF23, primarily mediated through Fam20 C-mediated phosphorylation, GALNT3-dependent glycosylation, and/or Furin-induced proteolytic processing. Nascent full-length iFGF23 is cleaved by intracellular proteases within FGF23-producing cells into cFGF23 and N-terminal fragments, which subsequently enter circulation alongside iFGF23 [16, 36–40]. This study systematically investigated the molecular mechanism underlying FG-4592-mediated reduction of iFGF23 levels in CKD. Remarkably, FG-4592 treatment induced an approximately two-fold upregulation of Furin mRNA expression in the bone tissue of CKD rats, without concurrent alterations in GALNT3 or FAM20 C expression levels, suggesting Furin plays a predominant role in FGF23 cleavage during FG-4592 treatment. These findings were subsequently validated through in vitro experimentation. Although GALNT3, FAM20 C, and Furin are all known to modulate FGF23 metabolism, our data reveal that FG-4592 specifically enhances Furin expression and activity without affecting GALNT3 or FAM20 C levels. This study is the first to demonstrate that FG-4592 reduces iFGF23 by promoting Furin-mediated cleavage, thereby clarifying a previously uncharacterized regulatory mechanism. Collectively, these data demonstrate that FG-4592 reduces circulating iFGF23 through enhanced Furin-dependent proteolytic processing.

Accumulating evidence suggests that, although the underlying pathogenesis remains incompletely elucidated, the kidney-bone axis, mediated by iFGF23, plays a pivotal role in CKD pathophysiology. Clinical studies have demonstrated that elevated circulating iFGF23 levels are independently associated with increased risks of all-cause and cardiovascular mortality, both in CKD patients and the general population, independent of traditional risk factors. This study provides indirect evidence that targeting iFGF23 represents a promising therapeutic strategy to attenuate renal fibrosis progression. Furthermore, the potential effects of FG-4592 on bone metabolism were investigated. To our knowledge, this study represents the first comprehensive characterization of this critical aspect. These findings provide a theoretical foundation for addressing clinical concerns regarding HIF-PHIs, including their effects on bone metabolism and vascular calcification. Previous studies indicated that vadadustat and molidustat, two other types of HIF-PHI, all significantly lowered plasma levels of iFGF23 and improved renal function in murine CKD models [11, 17]. Similarly, as reported by Megan et al., FG-4592 reduced iFGF23 in a CKD model [41]. However, the underlying molecular mechanism of effect of HIF-PHIs on FGF23 was not be elucidated. The innovative significance of our study lies in elucidating the specific molecular pathway through which HIF-PHIs ameliorate renal fibrosis. Our study for the first time demonstrated that HIF-PHIs attenuates fibrosis through inducing activation of the Furin-iFGF23 axis. Therefore, HIF-PHIs represent promising therapeutic candidates due to their pleiotropic effects on CKD–mineral and bone disorder (CKD-MBD) and disease progression.

The extensive role of HIF-1α activation in organ protection was supported by recent study. For instance, zaruba et al. [42]. found that Roxadustat significantly improved the cardiac function of mice after myocardial infarction and reduced the adverse left ventricular remodeling by up-regulating HIF-1α and its downstream cxcl12/cxcr4/ackr3 axis. This is consistent with the anti-fibrosis effect observed in CKD model, suggesting that HIF activation reduces the pathological remodeling of other organs. Of note, future study needs to explore the dynamic regulation network of HIF in different organs to optimize the treatment.

This study has some limitations. Firstly, dynamic assessment of FGF23 levels was not performed during the treatment period. Secondly, the clinical study was conducted in a single-center setting with a relatively small sample size and limited observation period. A study with a larger sample size may be necessary in the future.

**Fig. 7:**
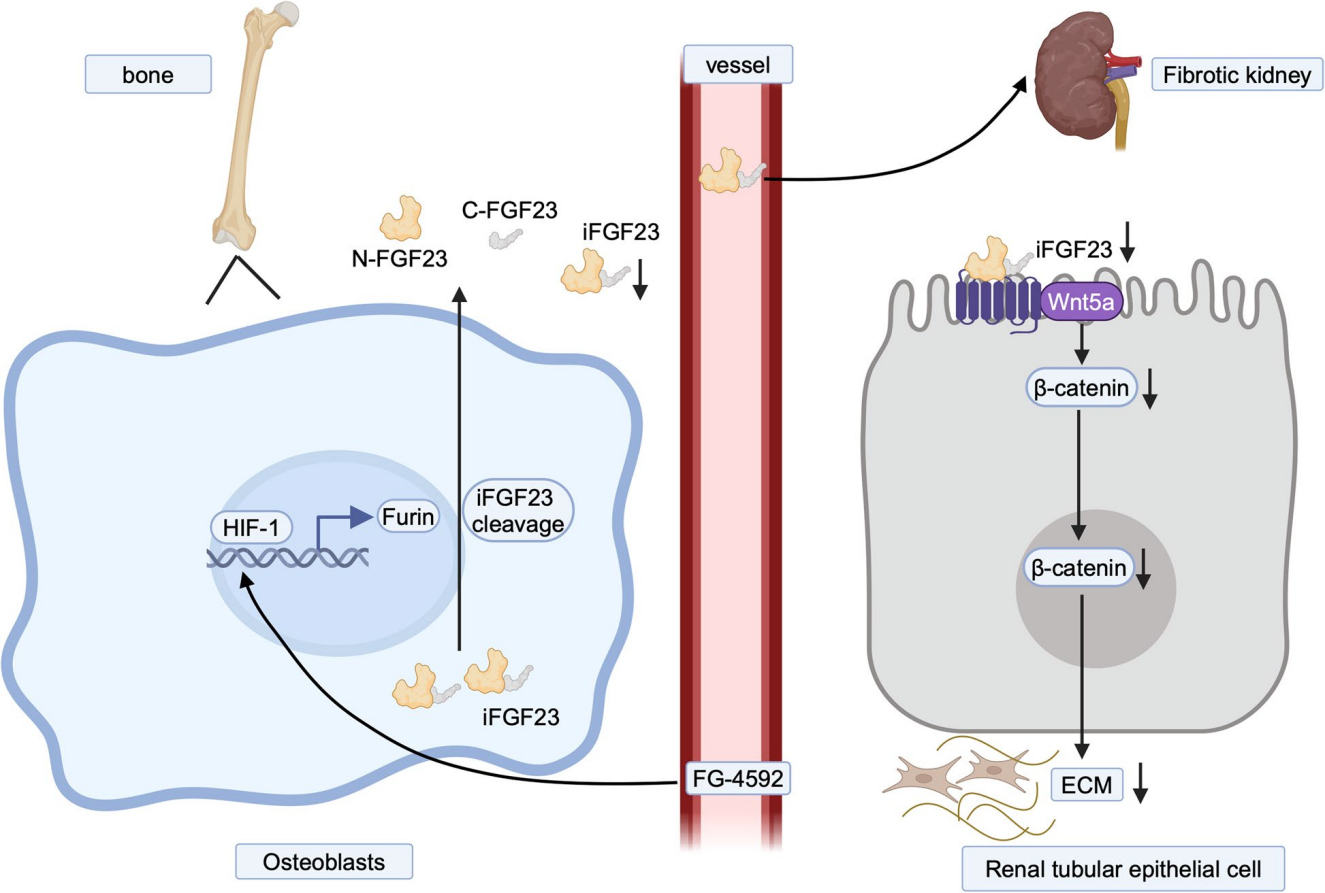
Schematic illustration of FG-4592 ameliorating TIF. During CKD, FG-4592 transcriptionally upregulates Furin expression, which subsequently cleaves iFGF23, leading to a reduction in iFGF23 levels and amelioration of TIF

In conclusion, we found that therapeutic-dose FG-4592 treatment could significantly lower iFGF23 and ameliorate TIF. Mechanistically, we demonstrated that inhibition of the iFGF23-WNT5A pathway is the exact mechanism by which FG-4592 ameliorated TIF. Further, we also demonstrated that the activation of Furin enzyme is the exact molecular mechanism for FG-4592-mediated iFGF23 cleavage (Fig. 7). These findings provide novel insights into the therapeutic potential of HIF-PHIs for ameliorating anemia and TIF in CKD, with implications for clinical practice. Notably, this study may pave the way for future research or clinical interventions in CKD patients.

## Supplementary Information

Supplementary Material 1.

## Data Availability

No datasets were generated or analysed during the current study.

## Abbreviations

TIF: Renal tubulointerstitial fibrosis
CKD: Chronic kidney disease
HIF-PHI: Hypoxia-inducible factors prolyl hydroxylase domain inhibitor
iFGF23: Intact FGF23
cFGF23: C-terminal FGF23
ChIP: Chromatin immunoprecipitation
Scr: Serum creatinine
BUN: Blood urea nitrogen
rFGF23: Recombinant FGF23
α-SMA: Alpha smooth muscle actin
TGF-β1: Transforming growth factor-β1
