# Supplementary material for: The Roxadustat (FG-4592) ameliorates tubulointerstitial fibrosis by promoting intact FGF23 cleavage
Source: Cell Commun Signal. 2025 Apr 25;23:200. doi: 10.1186/s12964-025-02175-2 (PMC12032739; doi:10.1186/s12964-025-02175-2)
Supplement: Supplementary file 1 — Supplementary Material 1. [file 12964_2025_2175_MOESM1_ESM.docx]

**Supplementary material**

**Supplementary Figure**


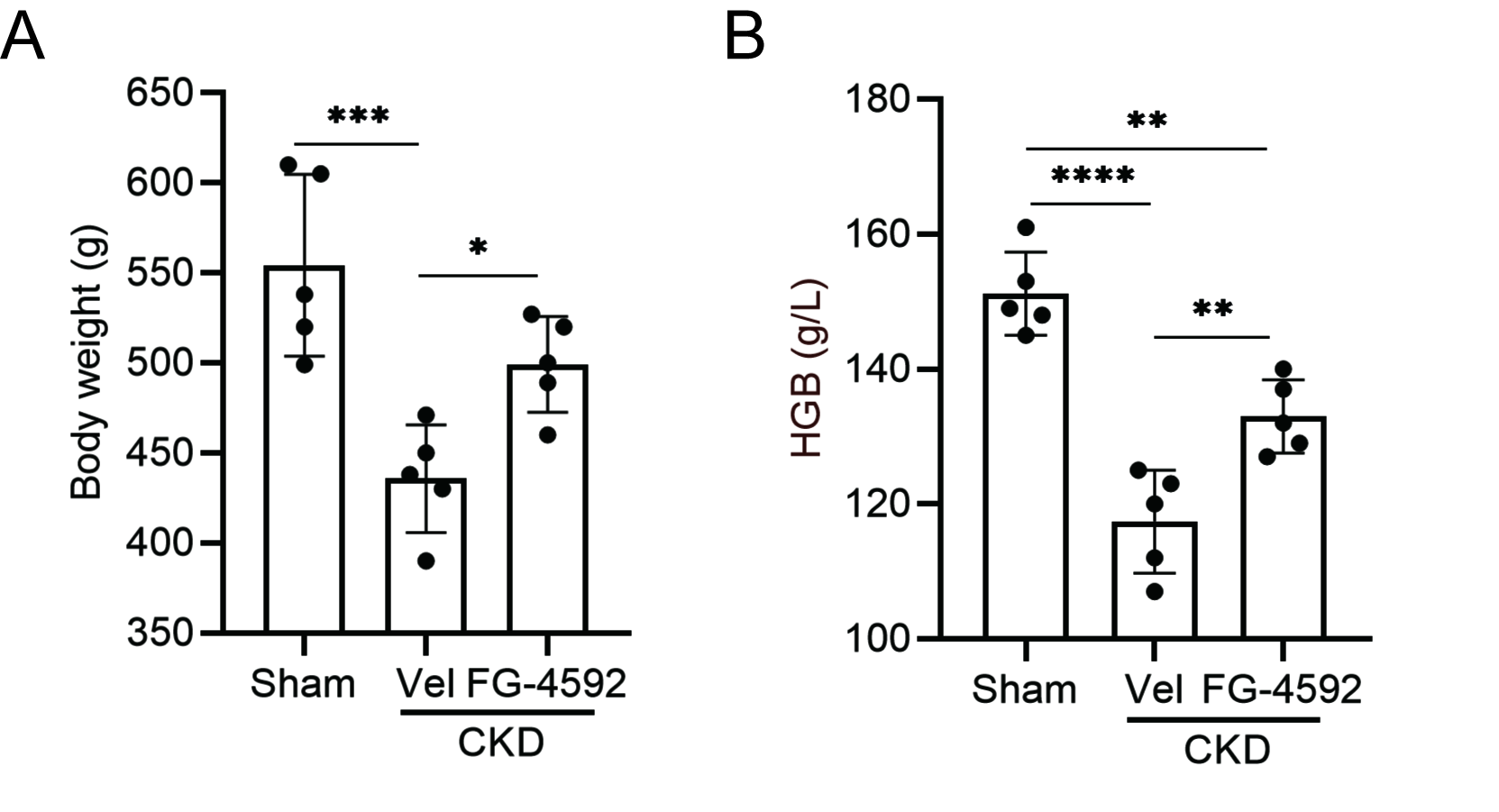


**Supplementary Figure 1.** **FG-4592 administration ameliorated anemia and attenuated body weight loss in rats.** Parameters measured include (A) The body weight of different groups of rats: Sham, CKD+vehicle, CKD+FG-4592. (B) HGB of different groups of rats. Data are presented as means ± SD (n=5). **p*<0.05, ***p*<0.01, ****p*<0.001, *****p*<0.0001.

**Supplementary Figure 2.** **Correlation between renal function and fibrosis indicators.** (A) Scatter plots with linear regression show significant correlation between BUN and Collagen 1 staining of the kidney in CKD rats. (B) Correlation analysis between Scr and Collagen 1 staining of the kidney in CKD rats. (C) Correlation analysis between BUN and α-SMA staining of the kidney in CKD rats. (D) Correlation analysis between Scr and α-SMA staining of the kidney in CKD rats. (E) Correlation analysis between BUN and Fibronectin staining of the kidney in CKD rats. (F) Correlation analysis between Scr and Fibronectin staining of the kidney in CKD rats.


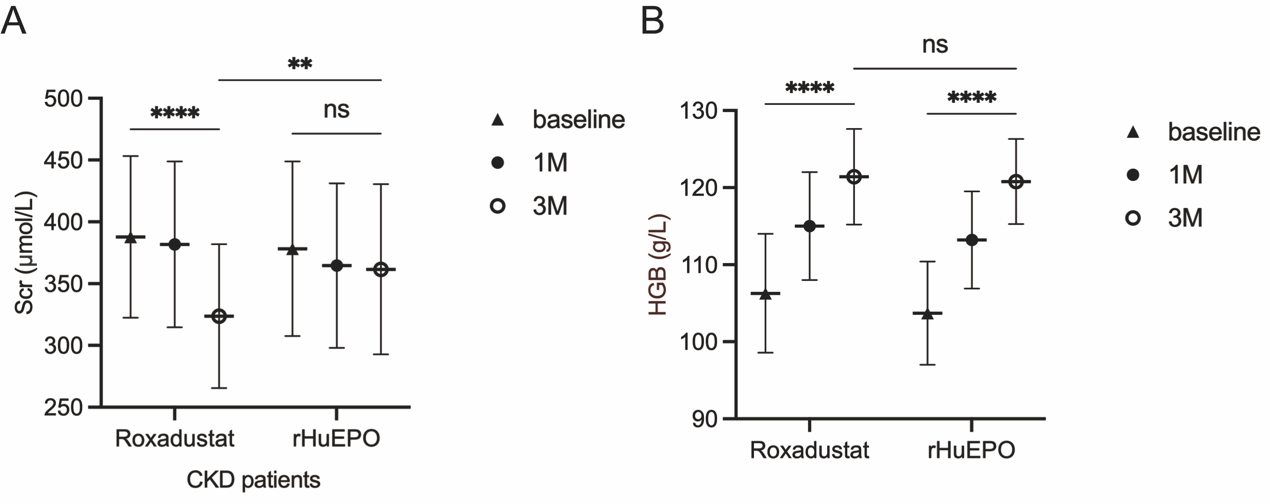


**Supplementary Figure 3. Comparison of Parameters Between the Roxadustat and Erythropoietin Groups at Baseline, 1 Month, and 3 Months Post-Treatment.** (A) HGB; (B) Scr.

**
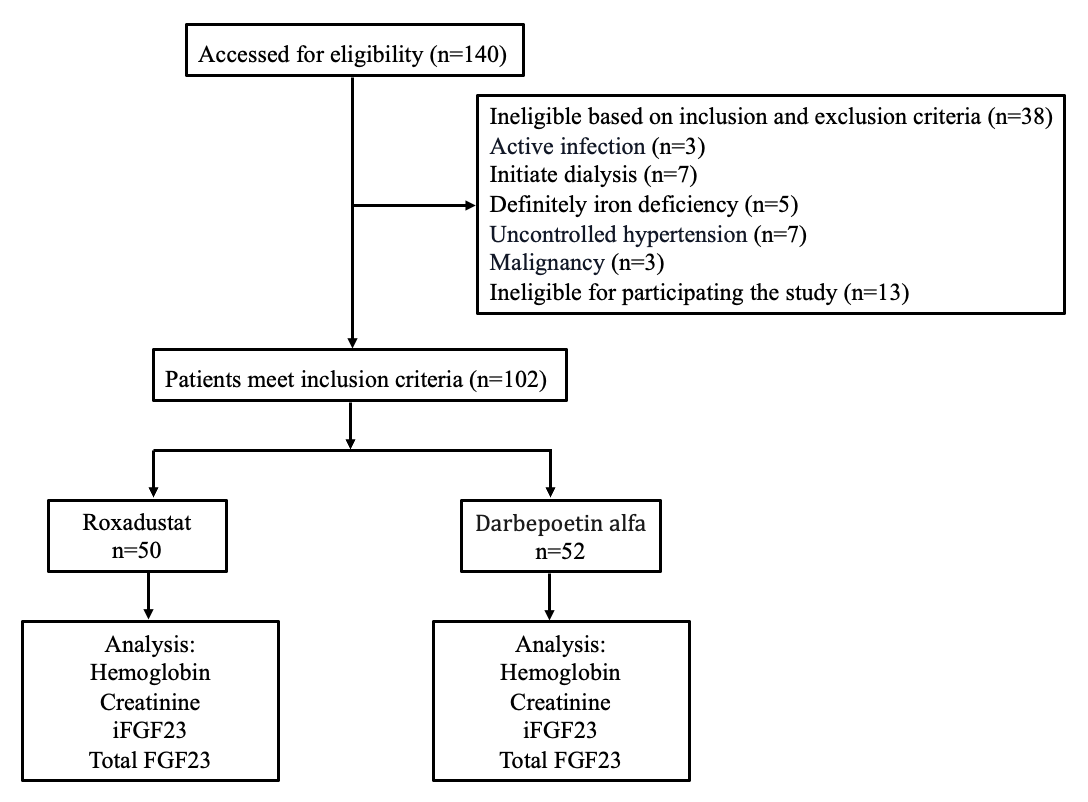
**

**Supplementary Figure 4. Flowchart of the study.**

**Supplementary Table**

**Supplementary Table 1. Primers for real-time quantitative PCR**

**Homo**

| *Gene* | *Forward (5’-3’)* | *Reverse (3’-5’)* |
| --- | --- | --- |
| *WNT5A* | *CTTGAGCACGACGAAGCAAC* | *GAGACAAAGGGGTGAGGCAG* |
| *COL1A1* | *GAGGGCCAAGACGAAGACATC* | *CAGATCACGTCATCGCACAAC* |
| *ACTA1* | *GGCATTCACGAGACCACCTAC* | *CGACATGACGTTGTTGGCATAC* |
| *TGFB1* | *GGCCAGATCCTGTCCAAGC* | *GTGGGTTTCCACCATTAGCAC* |
| *CTNNB1* | *GCTGGGACCTTGCATAACCT* | *CCAAGCATTTTCACCAGGGC* |
| *GAPDH* | *TCCAAAATCAAGTGGGGCGA* | *AAATGAGCCCCAGCCTTCTC* |

**Rat**

| *Gene* | *Forward (5’-3’)* | *Reverse (3’-5’)* |
| --- | --- | --- |
| *Tgfb1* | *GGCTGAACCAAGGAGACG* | *CATGAGGAGCAGGAAGGG* |
| *Col1a1* | *TGGAGAAGAAGGAAAACG* | *CCAGGACTGCCAGTGAGA* |
| *Fn1* | *TGGCAACTCAAACGGGGA* | *GGGGAAGTGGCACAAGGC* |
| *Acta2* | *ATTATGTTTGAGACCTTC* | *TCCAGCACAATACCAGTT* |
| *Fgf23* | *TATTCAGACACTTCCCCA* | *CCCCTATTATCACTACGG* |
| *Fam20c* | *AGCGGTGGACCTCTATCCTAACTG* | *CATGGCGACACTGGTGATCTTCTG* |
| *Hif-1α* | *CCGCCACCACCACTGATGAATC* | *CCGACTGTGAGTACCACTGTATGC* |
| *Gapdh* | *GGCTCTCTGCTCCTCCCTGT* | *CGTTCACACCGACCTTCACC* |
| *Furin* | *AATGACAACAGGCACGGCACTC* | *GTCACCTCGCCATCCAACATACG* |
| *Galnt3* | *ATAACCAGGGAGACAAAC* | *CTCGCTGAGCAGAATACT* |
| *Wnt5a* | *AGCCGAGAGACAGCCTTCAC* | *TCCTGCGACCTGCTTCATTG* |
| *Ctnnb1* | *GCGACTAAGCAGGAGGGAT* | *CCCACTTGGCACACCATCAT* |

**Supplementary Table 2. Patient characteristics and laboratory data at baseline.**

| **Parameter** | **Roxadustat**  (N=50) | **rHuEPO**  (N=52) | ***P*** |  |
| --- | --- | --- | --- | --- |
| Age (years) | 53.2 ± 7.1 | 51.7± 6.6 | 0.292 |  |
| Males [*n* (%)] | 27 (54.0) | 31 (59.6) | NS |  |
| Diabetes mellitus [*n* (%)] | 21 (42.0) | 26 (50.0) | NS |  |
| Hypertension [*n* (%)] | 37 (74.0) | 32 (61.5) | NS |  |
| CKD stage [*n* (%)] |  |  |  |  |
| 3 | 19 (38.0) | 15 (28.8) | NS |  |
| 4 | 24 (48.0) | 27 (51.9) | NS |  |
| 5 | 7 (14.0) | 10 (19.2) | NS |  |
| CKD aetiology [*n* (%)] | |  |  |  |
| Chronic glomerulonephritis | 21 (42.0) | 24 (46.2) | NS |  |
| Diabetic nephropathy | 16 (32.0) | 19 (36.6) | NS |  |
| Others | 8 (16.0) | 5 (9.6) | NS |  |
| Unknown | 5 (10.0) | 5 (9.6) | NS |  |
| Medications [*n* (%)] | |  |  |  |
| ARBs | | 36 (72.0) | 39 (75.0) | NS |
| Beta-blocker | | 31 (64.0) | 34 (65.3) | NS |
| Statins | | 24 (48) | 27 (51.9) | NS |
| Hemoglobin (g/L) | | 106.3 ± 7.7 | 103.7± 6.7 | 0.073 |
| Scr (μmol/L) | | 387.8 ± 65.3 | 378.3 ± 70.6 | 0.978 |
| iFGF23 (pg/mL) | 1428 ± 509.5 | 1353 ± 448.9 | 0.433 |  |
| Total FGF23 (pg/mL | 3426 ± 1345 | 3490 ± 1380 | 0.730 |  |

Scr, Serum Creatinine; iFGF23, intact FGF23; NS, no significant.

**Supplementary Table3. Comparison of Hemoglobin Between the Roxadustat and Erythropoietin Groups at Baseline and 3 Months Post-Treatment.**

| **Group** | **Hemoglobin (g/L)** | | |
| --- | --- | --- | --- |
|  | **baseline** | **3M** | ***P*** |
| **Roxadustat** | 106.3 ± 7.7 | 121.4± 6.2 | 0.292 |
| **rHuEPO** | 103.7± 6.7 | 120.8 ± 5.5 | 0.0001 |
| ***P*** | 0.4831 | 0.9982 |  |

**Supplementary Table4. Comparison of Scr Between the Roxadustat and Erythropoietin Groups at Baseline and 3 Months Post-Treatment.**

| **Group** | **Scr (μmol/L)** | | |
| --- | --- | --- | --- |
|  | **baseline** | **3M** | ***P*** |
| **Roxadustat** | 387.8 ± 65.3 | 323.7± 58.2 | 0.001 |
| **rHuEPO** | 378.3 ± 70.6 | 361.6 ± 68.8 | 0.794 |
| ***P*** | 0.978 | 0.047 |  |

**Supplementary Table5. Comparison of iFGF23 Between the Roxadustat and Erythropoietin Groups at Baseline and 3 Months Post-Treatment.**

| **Group** | **iFGF23 (pg/mL)** | | |
| --- | --- | --- | --- |
|  | **baseline** | **3M** | ***P*** |
| **Roxadustat** | 1428 ± 509.5 | 1071 ± 428.3 | 0.001 |
| **rHuEPO** | 1353 ± 448.9 | 1385 ± 412.3 | 0.999 |
| ***P*** | 0.963 | 0.008 |  |

**Supplementary Table6. Comparison of total FGF23 Between the Roxadustat and Erythropoietin Groups at Baseline and 3 Months Post-Treatment.**

| **Group** | **Total FGF23 (pg/mL)** | | |
| --- | --- | --- | --- |
|  | **baseline** | **3M** | ***P*** |
| **Roxadustat** | 3426 ± 1345 | 4368 ± 1707 | 0.020 |
| **rHuEPO** | 3490 ± 1380 | 3793 ± 1351 | 0.999 |
| ***P*** | 0.999 | 0.037 |  |
